# An efficient approach to isolate STAT regulated enhancers uncovers STAT92E fundamental role in *Drosophila* tracheal development

**DOI:** 10.1016/j.ydbio.2010.02.015

**Published:** 2010-04-15

**Authors:** Sol Sotillos, Jose Manuel Espinosa-Vázquez, Filippo Foglia, Nan Hu, James Castelli-Gair Hombría

**Affiliations:** a CABD, CSIC/Universidad Pablo de Olavide, Seville, Spain; b Department of Zoology, University of Cambridge, Downing Street, Cambridge CB2 3EJ, UK

**Keywords:** STAT gene-regulation, Trachea specification, Ventral veinless, Trachealess, Enhancer localization, Gene-desert

## Abstract

The *ventral veinless* (*vvl*) and *trachealess* (*trh*) genes are determinants of the *Drosophila* trachea. Early in development both genes are independently activated in the tracheal primordia by signals that are ill defined. Mutants blocking JAK/STAT signaling at any level do not form a tracheal tree suggesting that STAT92E may be an upstream transcriptional activator of the early trachea determinants. To test this hypothesis we have searched for STAT92E responsive enhancers activating the expression of *vvl* and *trh* in the tracheal primordia. We show that STAT92E regulated enhancers can be rapidly and efficiently isolated by focusing the analysis on genomic regions with clusters of putative STAT binding sites where at least some of them are phylogenetically conserved. Detailed analysis of a *vvl* early tracheal enhancer shows that non-conserved sites collaborate with conserved sites for enhancer activation. We find that STAT92E regulated enhancers can be located as far 60 kb from the promoters. Our results indicate that *vvl* and *trh* are independently activated by STAT92E which is the most important transcription factor required for trachea specification*.*

## Introduction

The JAK/STAT signaling pathway is conserved from vertebrates to invertebrates (Hou et al., 2002; Levy and Darnell, 2002). STAT proteins are transcription factors whose misregulation in humans has been associated to various diseases including cancer. In *Drosophila,* JAK/STAT signaling controls diverse developmental processes including sex specification, segmentation, organogenesis and stem cell regulation (Arbouzova and Zeidler, 2006; Hombría and Brown, 2002; Luo and Dearolf, 2001). The identification of the direct STAT targets is of great scientific and medical relevance as they will help understanding how STAT controls such varied processes. *Drosophila*, with its single STAT protein (STAT92E), offers an excellent model to study this issue. In mammals the seven STAT proteins preferentially bind sites containing the palindromic TTC*n*GAA sequence where *n* represents a number of bases from 2 to 4. The different vertebrate STATs show specific binding preferences: STAT6 mainly binds 4*n* sites, while most other STATs bind preferentially 3*n* sites although STAT3 can also bind 2*n* sites (Decker et al., 1997; Ehret et al., 2001; Kisseleva et al., 2002). *Drosophila* STAT92E shows 3*n* site binding preference (Yan et al., 1996). However, *in vitro* STAT92E also binds 4*n* sites with lower affinity and *in vivo* it can regulate target genes using both 3*n* and 4*n* sites (Rivas et al., 2008). Identifying the genes directly regulated by STAT92E can help to understand the general rules about how STAT proteins achieve transcriptional specificity. The expression of several genes has been reported to be under the regulation of STAT92E and the number keeps expanding through the use of genetic and genomic assays. However, only in few cases the regulation has been proved to be direct by mutating the STAT92E sites either in cell lines (SOCS36E, Raf) (Baeg et al., 2005; Kwon et al., 2000; Muller et al., 2005), or *in vivo* (*eve, crb*, *dome*) (Lovegrove et al., 2006; Rivas et al., 2008; Yan et al., 1996).

The *in vivo* confirmation that a suspected target is directly regulated by STAT92E is a laborious task requiring the identification of the STAT regulated enhancer in the gene of interest and the molecular confirmation of the putative STAT sites. In this work, using as a test case the regulation of the early *Drosophila* tracheal genes, we explore how comparative bioinformatics analysis of genomic regions can be used to rapidly identify STAT regulated enhancers.

The tracheal tree of *Drosophila* forms a complex reproducible network of tubes capable of directly delivering oxygen to the animal's cells. The trachea has been used as a model to study how different signaling pathways form such stereotypic network. The tracheae originate from 10 ectodermal placodes present at stage 10 (st10) on each side of the embryo (Manning and Krasnow, 1993). These clusters of about 80 to 90 epidermal cells are located in each segment from the second thoracic (T2) to the eighth abdominal segment (A8). Typically, each cluster invaginates to form the tracheal pit that then buds six branches that migrate in different directions. The branches migrating anteriorly and posteriorly fuse to the equivalent branches of the adjacent segments creating a continuous dorsal trunk that runs along the anterior–posterior axis of the embryo and connects in T1 and A8 to the spiracles allowing the gas exchange. The remaining branches target the diverse organs or fuse to other tracheal branches (Manning and Krasnow, 1993).

Genetic analysis has shown that many signaling pathways are required to pattern this stereotyped network. Among others the FGF, EGF, dpp/TGFß, the wg/wnt, Notch, hedgehog and the JAK/STAT pathways are involved in different aspects of trachea morphogenesis [reviewed in (Ghabrial et al., 2003)].

**Fig. 1:**
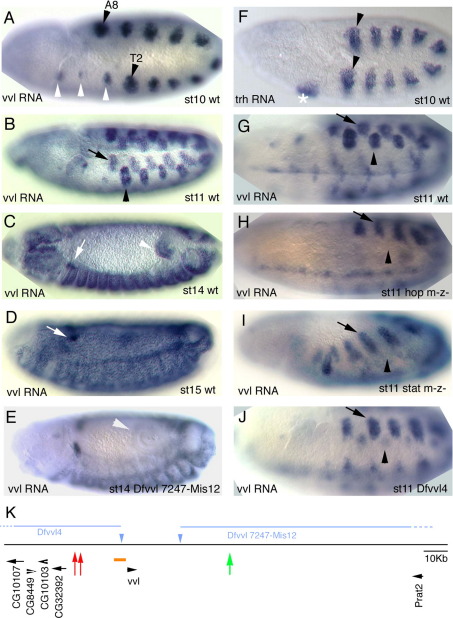
*vvl* expression in wild type and mutant backgrounds. All embryos show *vvl* RNA *in situ* expression except (F) that shows *trh* RNA expression. (A–D) Wild type *vvl* RNA expression. (A) At early st10 *vvl* is transcribed in the primordia of the 10 tracheal pits (black arrowheads in T2 and A8 mark the first and last primordia) and in homologous cells in segments that will not form trachea (white arrowheads). (B) At st11 10 dorsal epithelial patches (black arrow) appear in each trunk segment displaced with respect to the tracheal primordia (black arrowhead). (C) At st14 epithelial expression has spread to almost all the trunk although certain cells, like some in the posterior spiracles, never express *vvl*. The anterior hindgut (white arrowhead) also expresses *vvl* from st11. (D) At st15 high levels of *vvl* are expressed in the trachea and in a group of cells migrating dorsally (white arrows in C and D) that will join the ring gland. (E) *vvl* RNA *in situ* in a *Dfvvl 7247-Mis12* embryo lacking most of the *vvl* downstream region showing absence of hindgut expression (white arrowhead). (F) *trh* RNA *in situ* in a wild type st10 embryo showing expression in the salivary gland primordium (asterisk) and in the 10 tracheal pits (black arrowheads mark the first and last pit primordia). (G) Ventro lateral view of a wild type st11 embryo showing *vvl* expression in the tracheal pit (black arrowhead) and the dorsal patch (arrow). (H) In null *hop* mutant embryos at st11 *vvl* is not expressed in the tracheal pits (arrowhead), while the dorsal patches (arrow) and the midline expression develop normally. (I) Similarly, in null *stat92E* mutant embryos *vvl* is not expressed at st11 in the tracheal pits (arrowhead), while the dorsal patches (arrow) develop normally. (J) *Dfvvl4* embryos with deleted upstream region have no tracheal expression at st10 until at st11 they start developing very weak *vvl* expression in the tracheal pits (arrowhead). (K) Cartoon depicting the *vvl* genomic region. The intronless *vvl* transcript is located at 27 kb from CG32392 and at 117 kb from Prat2. The orange box represents the 7 kb area analyzed in previous works that uncovered the autoregulatory *RX-drf* enhancer. Red arrows mark the position of the early trachea enhancers, green arrow the position of the hindgut enhancer. Blue triangles represent the FRT piggyBac transposable elements used to delete the upstream and downstream *cis*-regulatory region (blue lines).

Despite much interest on trachea development little is known about the early tracheal placode specification. The earliest sign of tracheal development is observed at st10 (about 5 h of development at 25 °C) when thickenings of the dorsal epithelium form the tracheal placodes. Two genes encoding transcription factors expressed at this early stage are required for the normal trachea morphogenesis: *trachealess* (*trh*), encoding a bHLH-PAS protein (Isaac and Andrew, 1996; Wilk et al., 1996) and *ventral veinless* (*vvl*), encoding a POU-domain protein (Anderson et al., 1996; de Celis et al., 1995). The *vvl* and *trh* genes are the most upstream genes with tracheal specific expression (Fig. 1A,F) and their early activation is independent of each other (Boube et al., 2000). In *trh* mutant embryos the tracheal pits do not form and the tracheal precursors stay on the embryo's surface (Isaac and Andrew, 1996; Wilk et al., 1996). In *vvl* mutant embryos the tracheal pits invaginate but the cells do not branch appropriately (Anderson et al., 1995; de Celis et al., 1995). The *trh* and *vvl* early tracheal enhancers have not been identified and it is not yet known what are the upstream regulators controlling their activation in the tracheal primordium. Some reports suggest that the JAK/STAT pathway is the most upstream activator of *trh* expression in the tracheal placodes (Brown et al., 2001; Chen et al., 2002). Early *vvl* tracheal expression may be modulated by *wg*, *hh* and *dpp* but no direct molecular evidence has been provided (de Celis et al., 1995). *vvl* expression in the hindgut is activated downstream of JAK/STAT (Brown et al., 2003), but it is not known if *vvl* is also controlled by JAK/STAT in the trachea. Finding the *vvl cis*-regulatory regions is not trivial as the intergenic region is 144 kb long making it one of the largest in *Drosophila*. Here we test if a directed approach focusing on regions that contain conserved putative STAT92E binding-sites would help finding the enhancers without the need to methodically dissect the whole region. Using this approach we have isolated the *vvl* hindgut enhancer and two independent early trachea enhancers all of which depend on JAK/STAT activity. Molecular analysis of one of the early tracheal enhancers confirmed that *vvl* is directly regulated by STAT92E. Applying the same approach to the *trh* genomic region we have uncovered the STAT92E dependent trachea enhancers. We found that in both *vvl* and *trh* the STAT92E regulated enhancers are tens of kilobases away from the promoter. These results show that a targeted search of phylogenetically conserved STAT92E sites can be used efficiently to uncover enhancers dependent on STAT regulation in other genes. Our findings strongly suggest that the early expression of both main tracheal transcription factors is regulated directly by STAT92E underscoring the importance of the JAK/STAT pathway for trachea specification.

## Materials and methods

### Detection of *cis*-regulatory elements

We compared the genomic *vvl* and *trh* sequences of 10 *Drosophila* species including *Drosophila* *melanogaster*, *D. simulans*, *D. sechellia*, *D. erecta*, *D. yacuba*, *D. anannasae*, *D. grimshawi*, *D. virilis*, *D. mojavensis* and *D. persimilis*. Seven *vvl* regions containing conserved putative STAT92E DNA-binding sites were tested for their *cis*-regulatory activity. As a control we also chose three regions containing non-conserved STAT92E sites and two lacking STAT92E binding sites. Flanking primers (Supplementary Table 1) were used to amplify the *D. melanogaster* DNA and the fragments were first subcloned into pGEM-T easy (PROMEGA) and then transferred to a pCaSpeR with *lacZ* under the control of a minimal promoter (*phs43lacZ*). Using the same approach we tested eight *trh* genomic fragments containing conserved putative STAT92E sites. (For flanking primers see Sup. Table 2.) Constructs were injected in *D. melanogaster* by Bestgene (USA) and the *Drosophila* Consolider-Ingenio 2007 transformation platform (Spain). The resulting reporter genes were tested for expression staining with either anti-ßGal or with a *lacZ* RNA probe. For most enhancers at least four independent transgenic lines were analyzed, with most lines showing consistent patterns of expression (see Sup. Tables 4 and 5 to find the actual number of lines analyzed for each construct).

### Immunohistochemistry and RNA in situ hybridization

Embryos were fixed in 1:1 formaldehyde 4% in PBS/*n*-heptane for 20 min at room temperature and stained with mouse anti-ßGal to detect reporter gene expression (PROMEGA). Standard digoxigenin RNA in situ protocols were performed with *lacZ*, *trh* and *vvl* RNA probes. For double RNA *in situ* the *vvl* probe was labeled with fluorescein RNA labeling mix and the *upd* probe with DIG RNA labeling mix (Roche). Embryos were simultaneously hybridized at 55 °C overnight with both probes. We used anti-Fluorescein-POD (horseradish peroxidase) to detect the fluorescein labeled probe developing it with DAB and hydrogen peroxide, and then used anti-DIG-AP to detect the DIG labeled probe following the standard RNA *in situ* procedures. For fluorescent double *in situ*-antibody stainings embryos carrying the relevant reporter constructs were hybridized overnight with a *vvl* RNA probe. The embryos were incubated 4 h with mouse anti-ßGal and goat anti-DIG followed by an overnight incubation with anti-ßGal and anti-goat Alexa647. Finally, on the third day the embryos were incubated 3 h with anti-mouse Alexa448 and observed under confocal microscope.

### Mutation of STAT92E DNA-binding sites

Putative STAT92E sites in *vvl1+2* were mutated with QuikChange (Stratagene) using the oligos described in Supplementary Table 3.

### Generation of vvl *cis*-regulatory upstream and downstream deletions

We deleted the *vvl* upstream *cis*-regulatory region with the FLP-FRT technique (Parks et al., 2004) using *P[BacWH]f05223*, a piggyBac element inserted 4.5 kb upstream from the *vvl* transcription start, and *PBac[WH]f01945* that is inserted 74.5 kb upstream. The 70 kb deletion was confirmed by PCR amplification. Besides the upstream *vvl* sequence, this deficiency (named *Dfvvl4*) removes seven predicted genes. We also deleted part of the downstream *vvl cis*-regulatory region using the *PBac[WH]f07247* element, which is inserted 16 kb downstream of the *vvl* transcript and *PBac[WH]f03756* inserted 174 kb downstream of the *vvl* transcription unit*.* This deficiency, named *Df vvl 7247-Mis12*, generates a deletion of about 158 kb affecting 10 predicted genes.

### Genetic variants

We used the following null mutant alleles: *Df(1)os1A* (deficient for all upd ligands), and the amorphic alleles *hop*^*C111*^ (for *JAK*), *mrl*^*6346*^ (for *stat92E*), *vvl*^*GA3*^, *vvl*^*H599*^, *trh*^*8*^, *wg*^*CX4*^. Ectopic *upd* expression was induced expressing *UAS-upd* with the *69B-Gal4* line.

### Germ line clones

Null *hop* embryos were induced in *hop*^*C111*^ *FRT101*/*ovo*^*D1*^ *FRT101*; *hs-FLP/+* females by 1 h heatshock at second larval instar. The adult females were mated to either wild type, *vvlds1.5-lacZ*, *vvl1+2-lacZ* or *vvl345-lacZ* homozygous males. STAT92E amorph clones were induced in hsFLP/+; FRT82B *mrl*^*6346*^/FRT82B ovoD L2 females and mated to FRT82B *mrl*^*6346*^/TM6B males.

## Results

The *trh* gene is expressed and required for the specification of the salivary glands and the tracheal primordia (Fig. 1F, (Isaac and Andrew, 1996; Wilk et al., 1996)). The *ventral veinless* (*vvl*) gene of *Drosophila* is required for the development of several organs including the trachea, the midline glia, the chordotonal sensory organs and the wing (Anderson et al., 1995; de Celis et al., 1995; Inbal et al., 2003). In contrast to the stable *trh* expression during development, *vvl* has a very dynamic pattern (Fig. 1A–D). At stage 9 (st9) and early st10 expression first develops in the tracheal primordia (Fig. 1A) followed by expression in the midline glial precursors. At st11 dorsal ectodermal patches of expression appear out of register with the tracheal pits (arrows in Fig. 1B,G) and *vvl* expression appears in the anterior hindgut (not shown and Fig. 1C). As the embryos mature at st11 and 12, *vvl* ectoderm expression broadens up until at st13 it covers most epidermal cells from T1 to A9 (Fig. 1C). A few cells in the trunk remain void of *vvl* including some sensory organs in every segment and some cells in the anterior and posterior spiracles. At st14 the oenocytes (not shown) and a group of cells that will integrate into the ring gland show increased levels of *vvl* expression (arrows in Fig. 1C–D).

### *vvl* expression in the trachea and hindgut primordia depends on JAK/STAT function

Past work has shown that the JAK/STAT pathway is the earliest activator of *trh* in the tracheal primordia (Brown et al., 2001) but nothing is known about how the *vvl* early trachea expression is activated. As STAT92E is required for *trh* activation, and *trh* is necessary to maintain *vvl* expression from st11, a direct requirement of the JAK/STAT pathway for *vvl* tracheal activation is difficult to ascertain. To find out if JAK/STAT is required for early *vvl* activation, we carefully analyzed *vvl* expression in germ line mutants lacking either JAK or STAT92E before the Trh/Vvl cross-regulatory interactions start. We observe that in *stat92E* and *hop* germ line clone embryos (completely lacking STAT and JAK function respectively, see Materials and methods) *vvl* is not expressed in the tracheal placodes at st10 and early st11 (compare Fig. 1G and H–I), suggesting that *vvl* is also directly activated by STAT92E in the trachea. As previously reported (Brown et al., 2003), we also observed that the *vvl* expression in the hindgut requires JAK/STAT signaling.

**Fig. 2:**
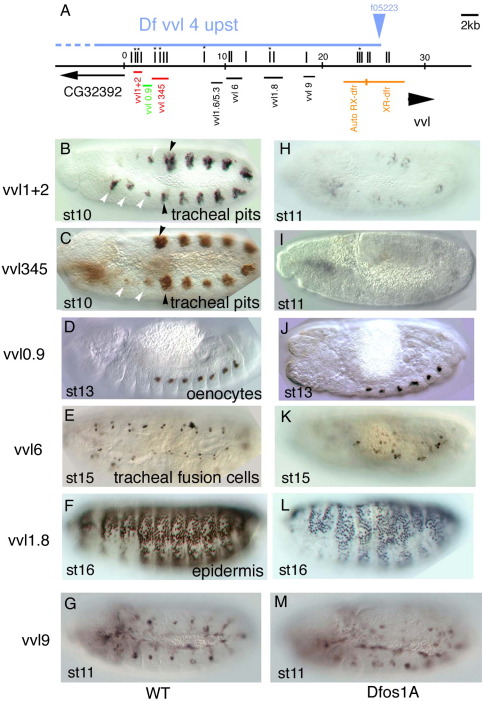
Upstream *vvl* embryonic enhancers in wild type and *upd* mutant background. (A) Cartoon showing the location of the upstream *vvl* enhancers analyzed. Vertical lines represent putative STAT92E binding sites. An asterisk marks sites conserved in all 10 studied Drosophilids. Orange line represents the upstream region analyzed previously (Certel et al., 1996). Red lines represent the early tracheal enhancers. Green line represents the oenocyte enhancer. Horizontal blue line indicates the deleted upstream region in Dfvvl4. (B–G) Expression of the indicated enhancers in wild type embryos. (H–M) Expression of the same enhancers in *Dfos1A* embryos lacking all three *upd* ligands. Note the strong effect that lack of *upd* has on *vvl1+2* and *vvl345* expression (H–I). Black arrowheads point to the tracheal pits, white arrowheads point to the homologous patches in anterior and posterior segments.

**Fig. 3:**
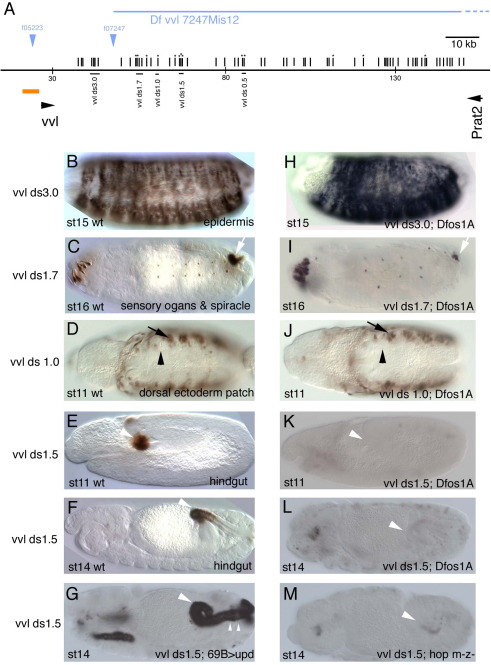
Downstream *vvl* embryonic enhancers in wild type and *upd* mutant backgrounds. (A) Cartoon representing the location of the downstream *vvl* enhancers analyzed. Symbols are as in Fig. 2. (B–F) Expression of the indicated enhancers in wild type embryos. (H–L) Expression of the same enhancers in a *Dfos1A* embryos lacking all three *upd* ligands. (K–L) Note the strong effect that lack of *upd* has on *vvlds1.5* hindgut expression. (G) Ectopic *upd* results in ectopic *vvlds1.5* expression in the most posterior area of the hindgut (small arrowheads) and in the salivary glands. (M) In null *hop* mutant embryos *vvlds1.5* is not expressed in the hindgut (arrowhead).

*vvl* is an intronless gene located in a 144 kb “gene-desert” region with the closest neighboring genes located 27 kb upstream and 117 kb downstream (Fig. 1K). In this large region only the immediate upstream 7 kb of *vvl cis*-regulatory DNA have been studied (Fig. 1K orange box) leading to the discovery of the autoregulatory *RX-drf* enhancer that becomes active at late st11 in the trachea, the oenocytes and the midline glia (Certel et al., 1996) (*drf* is a synonym for *vvl*). *RX-drf* expression is completely dependent on the endogenous Vvl protein expression (Certel et al., 1996; Zelzer and Shilo, 2000). Besides this autoregulatory module, no other tissue specific enhancers have been described in this vast intergenic region. To help localizing the *vvl* enhancers we generated deletions of the *vvl* upstream and downstream regions using the FLP-FRT technique (see Materials and methods). Embryos homozygous for the *Dfvvl4* upstream deletion (Figs. 1K and 2A) lack early st10 *vvl* expression in the tracheal pits, with *vvl* only appearing in the trachea at late st11 (Fig. 1J compare with G). These embryos form an aberrant tracheal tree that, as is the case in *vvl* null mutants, does not fuse in a longitudinal dorsal trunk (not shown). *vvl* expression in the ectoderm, hindgut, st11 dorsal ectoderm patches and midline glia is not affected in *Dfvvl4* (Fig. 1J and not shown) indicating that these enhancers lie downstream or are redundant localizing both in downstream and upstream regions. In contrast, embryos homozygous for the downstream deletion *Dfvvl7247-Mis12* (Figs. 1K and 3A) do not affect the trachea but lack hindgut expression (Fig. 1E).

### Search for STAT-regulated *vvl* enhancers

If *vvl* expression was controlled directly by STAT92E, the enhancers should contain STAT92E binding sites that would facilitate their location. We searched in the *vvl* genomic region for 3*n* (TTC*nnn*GAA) and 4*n* (TTC*nnnn*GAA) sequences that have been shown to be regulated by STAT92E *in vivo* (Rivas et al., 2008; Yan et al., 1996). These sequences are present at 85 places in the *vvl D. melanogaster* intergenic region (vertical stripes in Figs. 2A and 3A), 20 of which are conserved in the *vvl* genomic region of several Drosophilids (marked with an asterisk). We made 10 reporter genes containing the putative STAT92E sites and, as control, two lacking them testing their ability to express *lacZ* in *D.* *melanogaster* embryos (Sup. Table 4). Our analysis uncovered several embryonic enhancers upstream (Fig. 2B–G) and downstream (ds) (Fig. 3B–F) the *vvl* transcription unit. Among them two independent early (st10) trachea enhancers (Fig. 2B–C), and a hindgut enhancer active from st11 (Fig. 3E–F). We also identified other enhancers driving expression in sensory organs, ectoderm, late trachea (st13), posterior spiracles and oenocytes (Figs. 2 and 3 and Sup. Table 4). To confirm that the enhancers are expressed in the *vvl* pattern, we performed fluorescent *vvl* RNA *in situ* and ß-Gal double staining (Sup. Fig. 2). The ß-Gal expression in *vvl9* and *vvl1.0* was too weak and did not produce good double stainings. All other constructs, with the exception of the posterior spiracle enhancer, drive ß-Gal expression in *vvl* expressing cells.

As a rapid test for the requirement for JAK/STAT regulation, we analyzed the expression of these enhancers in *Dfos1A* embryos. This deficiency deletes *upd*, *upd2* and *upd3* that encode all *Drosophila* JAK/STAT ligands (Hombría et al., 2005). This showed that the early trachea and the hindgut enhancers require JAK/STAT function (Figs. 2H–I and 3K). Expression of *vvlds1.7* in the posterior spiracle is also lower in *Dfos1A* embryos than in wild type (Fig. 3C,I), however we believe this effect may be indirect as *vvlds1.7* is activated at st14 after *upd* transcription has stopped in the spiracle and there is no nuclear STAT92E in the posterior spiracle cells (Sotillos et al., 2008) and in any case this enhancer does not normally activate *vvl* transcription. In some cases a requirement of STAT92E to regulate the enhancers could not be ascertained due to the strong morphological defects caused by the absence of JAK/STAT function. For example, in *Dfos1A* embryos where the tracheal tree does not form, the absence of *vvl6* expression in the cells associated to the trachea could be an indirect effect due to the absence of trachea. Therefore, although we cannot discard these enhancers are regulated directly by STAT92E we will not consider them any longer.

### STAT92E regulation of the *vvl* hindgut enhancer

The *vvlds1.5* enhancer is among the fragments with more conserved putative *STAT92E* sites. *vvl* transcription in the anterior hindgut has been proposed to be under JAK/STAT regulation (Brown et al., 2003). The *vvlds1.5* enhancer correctly reports the *vvl* activation (Fig. 3E–F), however while *vvl* transcription in the hindgut stops at st15; *lacZ in situ* hybridization in *vvlds1.5* shows that this reporter is transcriptionally active up to st17 (not shown). Despite this anomalous behavior, the enhancer's regulation up to st15 mimics that described for the endogenous *vvl* transcription (Brown et al., 2003), with lack of Upd or JAK kinase resulting in lack of hindgut activation (Fig. 3K–M); and ectopic *upd* overexpression with *69B-Gal4* inducing *vvlds1.5* ectopic expression in the posterior hindgut (Fig. 3G). These data indicate a correlation between the accumulation of conserved putative STAT92E sites and the presence of enhancers whose expression depends on JAK/STAT activity.

### STAT92E regulation of early *vvl* trachea enhancers

**Fig. 4:**
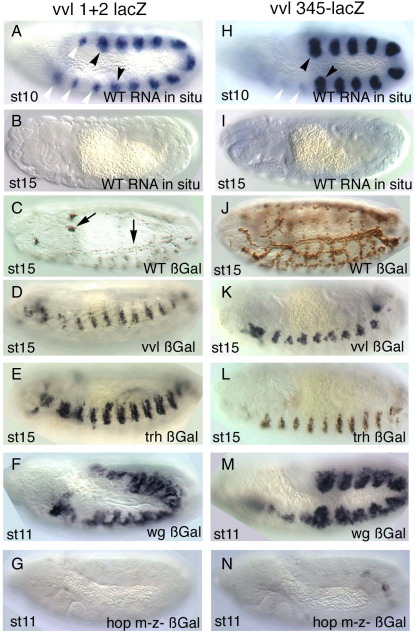
Expression of early *vvl* tracheal enhancers in various mutant backgrounds. (A–G) *vvl1+2* expression and (H–N) *vvl345* expression. (A) *lacZ* RNA *in situ* of a wild type *vvl1+2* st10 embryo showing the expression in the tracheal primordia (black arrowheads point the primordia in T2 and in A8) and in the anterior and posterior spots that will not form trachea (white arrowheads). (B) *lacZ* RNA *in situ* of a *vvl1+2* st15 embryo showing the enhancer is not expressed at later stages. (C) ß-Gal expression of a *vvl1+2* st15 wild type embryo. Note that long-lived ß-Gal perduring expression can be still detected in the trachea and in a group of cells moving dorsally (Arrows). (D) In *vvl* mutants *vvl1+2* is activated but the expressing cells do not form normal trachea. (E) In *trh* mutant embryos ß-Gal expressing cells do not invaginate staying on the embryo's epidermal surface. (F) In *wg* mutant embryos *vvl1+2* expression is not restricted to the tracheal primordia. Note that levels of expression are still lower outside the normal pit position. (G) *vvl1+2* expression is almost missing in *hop* mutants lacking maternal and zygotic JAK. (H) *lacZ* RNA in situ of a *vvl345* st10 embryo showing the expression in the tracheal primordia (black arrowheads point the primordia in T2 and in A8). The expression in the homologous spots in anterior segments is very weak (white arrowheads). (I) *lacZ* RNA in situ of a *vvl345* st15 embryo showing that the enhancer is not expressed any longer. (J) ß-Gal expression of a *vvl345* st15 embryo. The perduring protein can be detected by the antibody. (K) In *vvl* mutants *vvl345* is activated but the invaginated cells do not fuse into a tracheal tree. (L) In *trh* mutants *vvl345* is expressed but the cells remain on the embryonic epidermis. (M) In *wg* mutants *vvl345* expression is not restricted to the tracheal pits. (N) *vvl345* expression is almost missing in *hop* mutants lacking maternal and zygotic JAK. (A–B, H–I) show *lacZ* RNA *in situ*. (C–G, J–N) show anti-ßGal.

To confirm that JAK/STAT regulates early *vvl* tracheal expression we further analyzed the *vvl1+2* and *vvl345* trachea primordia enhancers. Analysis of the expression of both early enhancers by *lacZ in situ* shows that they drive expression very transiently at st10–st11 (Fig. 4A–B,H–I). The *vvl1+2* fragment drives *lacZ* expression in the tracheal placodes and in smaller spots in homologous regions of segments not forming trachea (Fig. 2B arrowheads). This pattern resembles the expression of *vvl* at st10 (Fig. 1A and de Celis et al., 1995). The three anterior spots correspond to the maxillary, labial and first thoracic segments while the two posterior spots correspond to the abdominal 9 and 10 segments (A9–A10). The *vvl345* fragment also drives expression in the trachea from stage 10 (Figs. 4H and 2C). The main difference with the *vvl1+2* fragment expression being that the small spots anterior and posterior to the tracheal pits are not labeled prominently.

To define at what level of the trachea morphogenetic cascade the *vvl1+2* and *vvl345* enhancers are acting, we studied if their expression is affected in mutants for *vvl* and *trh*, the earliest known tracheal specific genes (reviewed in Ghabrial et al., 2003). In *vvl* mutant embryos neither construct was affected (Fig. 4D,K) and we could observe that the labeled cells invaginated but were unable to form a normal tracheal network. These results prove that *vvl1+2* and *vvl345* are not autoregulatory enhancers.

As the maintenance of *vvl* expression in the trachea also requires Trh function (Boube et al., 2000; Zelzer and Shilo, 2000) we tested the expression of *vvl1+2* and *vvl345* in *trh* mutants. Both enhancers are activated normally in *trh* mutant embryos but the labeled cells do not invaginate, remaining on the surface of the embryo (Fig. 4E,L). To discard a redundant function of *trh* and *vvl* on the activation of the early trachea enhancers we also studied their expression in *trh*, *vvl* double mutant embryos and observed that even in this condition *vvl1+2-lacZ* and *vvl345-lacZ* are still expressed (not shown). The above results indicate that the early *vvl* trachea enhancers are controlled by the exogenous cues that define the early tracheal specification.

The WNT pathway has been suggested to be one such exogenous cue. Wg is expressed in every segment along a dorso-ventral stripe of cells running between the tracheal pits. Inactivation of the WNT pathway results in ectopic *vvl* expression in the areas between pits indicating that the pathway represses *vvl* transcription (de Celis et al., 1995). In *wg* null mutants, we observed ectopic expression of both *vvl1+2* and *vvl345* (Fig. 4F,M) indicating that *vvl* repression between pits may be mediated through these enhancers. As it also happens with the *vvl* RNA, the levels of expression in the tracheal pits are still higher than between pits suggesting that other negative elements besides the WNT pathway are modulating the expression.

As shown above the early *vvl* trachea enhancers are not expressed in *Df(1)os1A* embryos (Fig. 2H–I) suggesting that the JAK/STAT pathway is the positive exogenous cue activating *vvl*. However, as this mutation deletes other genes beside the *upd* ligands we confirmed the JAK/STAT dependence removing the JAK kinase by generating null *hop*^*C111*^ germ line clones embryos (Binari and Perrimon, 1994; Perrimon and Mahowald, 1986). As expected neither enhancer was expressed in *hop*^*C111*^ embryos lacking the maternal and zygotic *hop* gene (Fig. 4G,N).

### The early *vvl* trachea expression is regulated through the putative STAT92E binding sites

STAT92E mutants blocking the JAK/STAT pathway at any level of the signaling cascade do not form a tracheal tree, a phenotype that previously has been explained by their abnormal *trh* expression (Brown et al., 2001). However, the results here presented indicate that *vvl* is also regulated directly by the JAK/STAT pathway.

**Supplementary Fig. 1:**
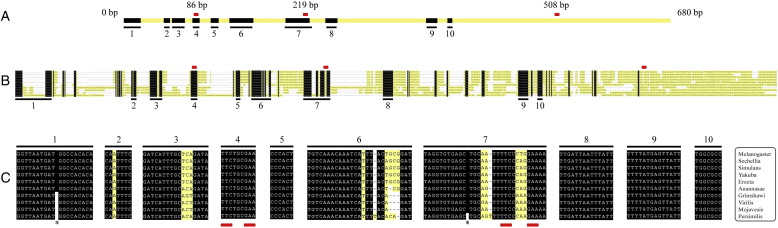
Conservation of the *vvl1+2* enhancer. (A) Schematic representation of the *vvl1+2* enhancer showing as black boxes the regions of sequence conserved in all 10 *Drosophila* species analyzed. Small red rectangles represent the three *D. melanogaster* STAT92E sites. Conserved boxes are numbered to relate them to panels B and C. Most homology blocks are grouped in two thirds of the fragment. (B) Alignment of the *vvl1+2* fragment in 10 *Drosophila* species. Black boxes represent regions of absolute sequence conservation, while non-conserved bases are represented in yellow. Note that some of the conserved blocks in *D. melanogaster* are split in some species and are separated in the figure by white gaps (-). (C) Sequence of the longest conserved blocks shown in A and B. Asterisks mark the location of gaps in some species. Only two STAT92E sites are in conserved sequence blocks. In this area we did not find any other STAT92E sites in other species.

**Supplementary Fig. 2:**
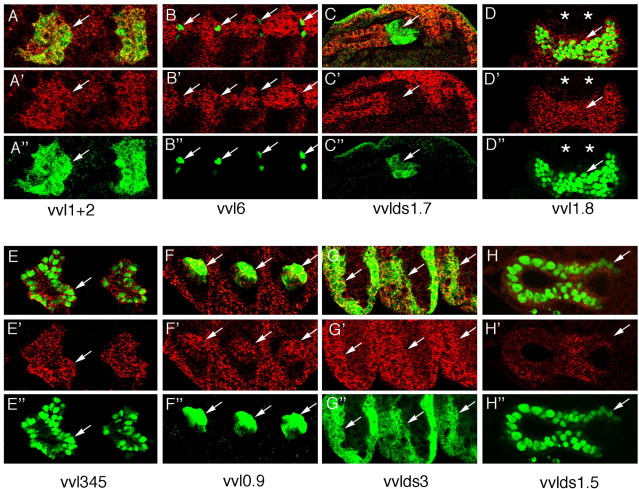
Expression of various enhancers in cells transcribing *vvl*. Top panels show close ups of double stained embryos of the indicated enhancer constructs labeled with anti-ßGal (green) and *vvl* RNA *in situ* (red). Middle panels (′) show *vvl* RNA. Lower panels (″) show ß-Gal staining. With the exception of *vvl 1.7* all enhancers drive expression in cells transcribing *vvl*. Arrows point to tracheal pits (A and E), isolated trachea cells that could represent fusion cells (B), spiracular chamber cells of the internal posterior spiracle (C), epidermal cells (D and G), oenocytes (F) and posterior hindgut (H). Note in H that both the *vvlds1.5* reporter construct and the *vvl* RNA are expressed in a gradient with lower levels in the posterior hindgut. Panel D is a dorsal view of A8 showing that the posterior spiracles (*) do not express *vvl*. All other panels show lateral views with anterior left and dorsal up.

**Fig. 5:**
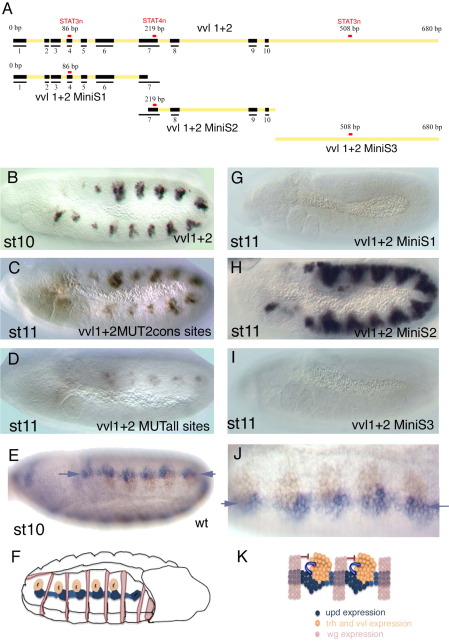
Expression of the *vvl1+2* early tracheal enhancers is controlled by STAT92E signaling. (A) Scheme of *vvl1+2* and subfragments tested. Black boxes represent blocks of conserved sequence in all 10 *Drosophila* species analyzed (see supplementary Fig.1 for sequence). Red rectangles represent the location of the three putative STAT92E DNA-binding sites. Only two of the sites are located in conserved blocks while the third one is only present in *D. melanogaster*. (B) Wild type *vvl 1+2* expression at st10. (C) ß-Gal expression driven from a *vvl 1+2* construct where the two conserved putative STAT92E binding sites have been mutated. Expression is lower than in the wild type, especially anterior to the second abdominal segment, but substantial levels of expression remain. (D) ß-Gal expression driven from a *vvl 1+2* construct with all three putative STAT92E binding sites mutated. Expression is highly reduced but traces still remain. (G) The *vvl1+2 MiniS1* fragment does not drive tracheal expression. (H) *vvl MiniS2* drives tracheal expression in embryos at st11. The expression seems to be less restricted with some ectopic signal between the pits. (I) The *vvl1+2 MiniS3* fragment does not drive tracheal expression. (E, J) A wild type st10 embryo double stained to show the *upd* RNA expression (purple) and the *vvl* RNA expression (brown). (J) is a magnification of E that has been rotated 180 degrees to have dorsal up and anterior left. Note the continuous *upd* stripe (purple arrows) running along the antero-posterior axis of the embryo just ventral to the *vvl* tracheal expression. (F) Scheme of the embryo shown in E that has been rotated 180° like panel J to have dorsal up and anterior left in the region of interest. Besides the tracheal pits and *upd* expression the scheme shows the location of the *wg* expressing cell stripes. (K) Scheme of proposed negative and positive regulation of early enhancers in the tracheal placodes. (B–D, G–I) Embryos are stained with anti-ßGal antibody. (E, J) show *upd* RNA in purple and *vvl* RNA in brown.

To confirm that the early tracheal enhancers are regulated through the putative STAT92E DNA-binding sites, we have analyzed the *vvl1+2* fragment that reflects the early *vvl* expression. Comparison of this 680 bp DNA fragment between several species of Drosophilidae (Stark et al., 2007) showed 10 conserved sequence blocks in the distal two thirds of the element (Fig. 5A and Sup. Fig. 1). Of the three putative STAT92E binding sites [TTC*nnn*(*n*)GAA] in the *vvl1+2* fragment two of them, a 3*n* and a 4*n* site, are conserved in all species while a further 3*n* site is only present in *D. melanogaster*.

We tested the effect of mutating some combinations of the putative STAT92E binding sites in the *vvl1+2* enhancer. Single mutation of the conserved 3*n* site at position 86 in *vvl1+2* does not affect tracheal expression significantly (not shown). Surprisingly, simultaneous mutation of the two conserved STAT92E sites at positions 86 and 219 only resulted in a mild decrease of the tracheal pit expression that is more noticeable in the segments with lower levels of expression (Fig. 5C). Mutation of all three sites reduces the expression from all segments (Fig. 5D).

Subdivision of the vvl1+2 element into three subfragments each containing a STAT92E site (Fig. 5A) shows that the two flanking fragments (*vvl1+2MiniS1* and *vvl1+2MiniS3)* cannot activate tracheal expression (Fig. 5G,I). In contrast, the central fragment (*vvl1+2MiniS2*) can drive tracheal expression although its spatial restriction is less defined with some embryos showing ectopic expression between the pits suggesting the loss of some repressor-binding site in the deleted region (Fig. 5H). These results show that the non-conserved *vvl1+2MiniS3* fragment is dispensable and cannot drive tracheal expression by itself. However, in the context of *vvl1+2*, when the conserved STAT92E sites are mutant, the non-conserved site at position 508 can contribute to enhancer activation. Thus a non-conserved site in a STAT92E site cluster can contribute to the enhancer's expression probably by interacting with other proteins binding to the enhancer core (vvl1+2MiniS2) that provide the tracheal activation specificity.

As we have seen, in *wg* mutant embryos, *vvl* and the early tracheal enhancers are expressed in a continuous band of cells running along the antero-posterior axis (Fig. 4F,M). These results indicate that the most upstream activator of *vvl* must be capable of activating *vvl* in the areas between the tracheal pits. Both *upd* and *upd2* are expressed in the tracheal pits at st11 (Harrison et al., 1998; Hombría et al., 2005) but the expression of *upd* with respect to the trachea primordia before the definition of the tracheal pits has not been analyzed carefully. Double RNA *in situ* of *upd* and *vvl* shows that the earliest activation of *vvl* occurs when *upd* is transiently expressed in a stripe that runs along the antero-posterior axis of the embryo just ventral to the tracheal placodes (Fig. 5E,J). Thus the pattern of expression of *upd* is consistent with that expected for the *vvl* trachea activator.

### Search of STAT92E regulatory elements in *trh*

**Fig. 6:**
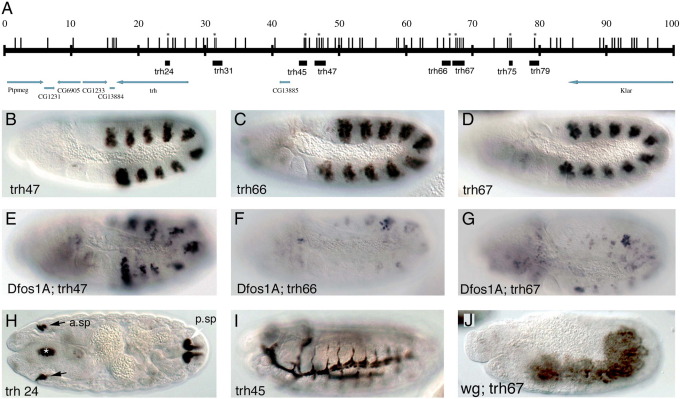
*trh* embryonic enhancers in wild type and mutant backgrounds. (A) Cartoon representing the location of the *trh* enhancers analyzed. Vertical lines represent putative STAT92E binding sites. An asterisk marks sites conserved in all Drosophilids. Black boxes represent the analyzed regions. Arrows indicate transcribed regions. Note that *CG13885* nests inside the *trh cis*-regulatory region. (B–D) Expression of three early *trh* tracheal enhancers in a wild type background. (E–G) Expression in *Dfos1A* embryos of the same enhancers shown in (B–D). Most expression disappears from *trh66* and *trh67* while substantial signal is still present in *trh47*. (H) Posterior (white arrow) and anterior spiracle (black arrows) expression driven by *trh24*, there is also some expression in the pharyngeal ectoderm (asterisk). (I) Expression of the *trh45* late trachea enhancer. (J) Expression of *trh67* in a *wingless* mutant background. Lateral views with head left dorsal up in all embryos except H that shows a frontal section.

**Fig. 7:**
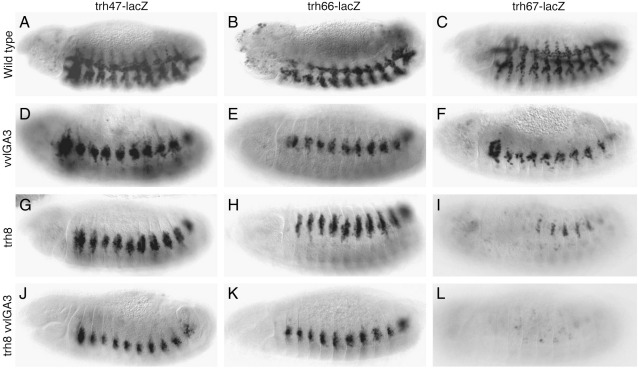
Expression of early *trh* tracheal enhancers in various mutants. (A–C) ß-Gal expression of the early enhancers at st13, all lines delineate the internalized tracheal network. (D–F) Expression of the early *trh* trachea enhancers is not affected in homozygous *vvl* mutant embryos despite the aberrant migration of the tracheal cells. (G–H) Expression of the *trh47* and *trh66* is maintained in *trh* mutant embryos with the uninvaginated ectodermal cells remaining on the embryo surface. (I) Expression of the *trh67* enhancer requires *trh* function suggesting a strong degree of autoregulation. (J–L) Expression of *trh47* and *trh66* in double *vvl trh* mutant embryos is maintained, while *trh67* is absent. Embryos in panels A–I are homozygous for the reporter constructs while in panels J–L are heterozygous.

Our results suggest that a targeted phylogenetic approach could be used to find STAT92E regulated enhancers. To prove the case for other putative STAT92E targets we tested if a similar approach could help finding the early *trh* trachea enhancers. We first analyzed the approximately 25 kb comprised between *CG13884* and *CG13885* including the *trh* introns (Fig. 6A). In this area there are only two regions with conserved putative sites one of which, *trh24*, drives expression in the anterior and posterior spiracles of the embryo and the other, *trh31*, is not expressed (Fig. 6H, and not shown). We observed that further upstream of *trh*, distal to *CG13885*, there is a gene-desert region of 40 kb with a high number of putative STAT92E sites. Given the precedent of the distal location of the trachea *vvl* elements we wondered if the trachea enhancers could locate here. We tested six fragments containing the conserved STAT92E sites and found that while the most distal two fragments, *trh75* and *trh79*, are silent the other four are expressed at different stages of trachea development. *trh47* and *trh66* are expressed in the trachea from st10, *trh67* from st11 and *trh45* from st13 (Fig. 6B–D and I). Of the three early trachea enhancers, RNA *in situ* with *lacZ* probes reveals that *trh47* and *trh66* are transiently expressed from st10 to st11 while *trh67* expression lasts up to st15 (not shown). To find at what level of the trachea cascade the three early enhancers are regulated we analyzed their expression in *Dfos1A*, *vvl*, *trh* and *vvl trh* double mutants. The expression of all three early enhancers is affected to different degrees in *Dfos1A* mutant embryos (Fig. 6E–G) and are ectopically expressed in *wg* mutants (Fig. 6J and not shown). Expression of the early enhancers does not require *vvl* (compare Fig. 7A–C with D–F). Only the expression of the *trh67* st11 enhancer is affected in *trh* and in the double *trh*, *vvl* mutants (Fig. 7G–L). Therefore, our results indicate that the *trh67* st11 enhancer is autoregulatory, while the st10 elements depend on upstream extrinsic tracheal signals. While the effect of the lack of *upd* ligands on *trh67* could be indirect due to the abnormal activation of *trh* in *Dfos1A* embryos, the effect on *trh66* may be direct. Surprisingly, the expression of *trh47* is not very abnormal (Fig. 6E) leaving open the possibility that there exists a still unknown exogenous tracheal activator.

Thus, by exclusively analyzing the conserved STAT92E sites in the *trh* genomic region we were able to uncover the early STAT92E regulated enhancers. It is interesting to note that there are conserved STAT92E sites associated to late tracheal enhancers. Upd expression is maintained on the trachea during most of embryogenesis (Harrison et al., 1998). The association of conserved STAT92E sites to the later enhancers suggests that Upd may also contribute to maintain *trh* expression in the trachea through these elements, however the previous requirement of STAT92E for early *vvl* and *trh* activation and the effects these have on trachea development do not allow reaching any conclusions on this respect. Taken together, these results confirm that a phylogenetically targeted approach is an efficient method to isolate the STAT92E regulated enhancers.

## Discussion

Mutations in STAT proteins are linked to disease in humans and in *Drosophila* they affect the development of several major organs, embryo segmentation and maintenance of gonad and gut stem cells. Finding the direct targets of STAT holds the key to understanding how such different functions are controlled. Microarray techniques can be used to analyze at the genome level genes that are up- or down-regulated by STAT while ChIP-chip analysis can identify DNA regions directly bound by STAT uncovering hundreds of possible targets. However, as the transcriptional up- or down-regulation detected in microarrays could be indirect, and STAT92E binds chromatin without necessarily resulting in transcriptional regulation (Shi et al., 2008), these results have to be validated by time-consuming *in vivo* experiments. In this work we show that analysis of STAT92E conserved sites provides a rapid way to isolate STAT92E regulated enhancers even in large *cis*-regulatory regions.

### Direct analysis of phylogenetically conserved STAT92E sites successfully uncovers JAK/STAT regulated enhancers

Mutations in the JAK/STAT pathway result in embryos only forming residual trachea fragments. This is caused by the abnormal activation of the early tracheal genes *trh* and *vvl* (this work and Brown et al., 2001; Chen et al., 2002) suggesting that the early trachea enhancers may be directly regulated by STAT92E in which case the trachea enhancers would be associated to STAT92E binding sites.

To test this we first localized *in silico* the putative STAT92E binding sites in the *vvl* 144 kb intergenic region (Rivas et al., 2008; Yan et al., 1996). We then compared *in vivo* the enhancer activity of regions that either (1) contain putative STAT92E sites that are conserved in several *Drosophila* species; (2) contain non-conserved putative STAT92E sites; or (3) contain no putative STAT92E sites. Of the 12 reporter lines made 10 have enhancer activity consistent with harboring embryonic *vvl cis*-regulatory elements. The expression of the enhancers either lacking STAT92E sites or containing non-conserved STAT92E sites is independent of JAK/STAT function. In contrast, of the seven *vvl* enhancers containing conserved sites, the expression of three of them required JAK/STAT regulation in the embryo. One of these enhancers drives expression in the hindgut and two are expressed in the trachea at st10. These results suggested that exclusively looking for conserved STAT92E sites would be sufficient to localize the STAT92E regulated sites, a prediction we have confirmed by isolating the *trh* gene tracheal enhancers. Although we cannot discard the presence of cryptic STAT binding sites that diverge from the ideal consensus we used in our analysis, such elements will probably have a minor contribution as ignoring their existence allowed us finding the main regulatory elements. Analysis of only eight fragments comprising a seventh of the *trh* locus was sufficient to find the early tracheal STAT92E responsive elements. This analysis uncovered that the locus extends at least 40 kb upstream the *trh* promoter, with some enhancers located beyond the first predicted neighboring gene.

In both the *trh* and *vvl* genes we found that other late tracheal enhancers are associated to STAT92E conserved sites suggesting that the STAT92E protein may be repeatedly used to control tracheal gene expression during development. This possibility is backed by the fact that *upd* is transcribed up to st13 and phosphorylated STAT92E can be detected in the trachea well after the st10 early specification stage (Harrison et al., 1998; Li et al., 2003). This late trachea specific expression of *upd* depends on *trh* (Bridget Lovegrove and JC-GH unpublished) suggesting that a feed-back loop maintains JAK/STAT activity during tracheal development.

We confirmed that the expression of the *vvl1+2* early tracheal enhancer depends on STAT92E by mutating its putative STAT92E binding sites. Our results show that mutation of all three putative STAT92E sites in the *vvl1+2* enhancer causes a severe loss of expression, indicating that we isolated a bona-fide direct enhancer. However, other possible direct enhancers like the *vvlds1.5* hindgut element show an unexpected behavior. While *vvlds1.5* perfectly recapitulates the *vvl* early hindgut activation its expression does not stop at st15, but keeps transcribing *lacZ* up to st17 well after the endogenous *vvl* gut transcription ceased. Despite this abnormal behavior, and pending direct site mutagenesis confirmation, we believe that our experiments show convincingly that the analysis of the genomic regions containing conserved STAT binding site clusters is an efficient way to quickly identify direct STAT92E regulated enhancers.

An important finding of our analysis has been the observation that STAT regulated enhancers can be tens of kilobases away from the transcriptional start of the target gene, even separated by another predicted gene. This indicates that STAT regulated enhancers can be functional at great distances and that search of STAT binding sites should not be restricted to the immediate vicinity of a gene.

### Conserved STAT92E site clustering

Assuming there was no base content bias, the probability of finding in the genome either a 3*n* or a 4*n* STAT92E site by chance is (2 × 1/4^6^) which is close to one site every 2 kb. In the 144 kb *vvl* genomic region there are 85 putative STAT92E sites, that is, 15 more than the expected 70 sites. Similarly, in the 70 kb *trh* locus there are 53 putative sites, 19 more than the expected 34 sites. The excess of sites found can be partially explained by selective pressure as there are 20 conserved sites in *vvl*. However, in the *trh* locus the 9 conserved STAT92E sites represent only half of the observed excess. An additional explanation for the excess STAT92E sites could be provided by the observation that in the regions were we found evidence for JAK/STAT regulation, there are clusters of conserved and non-conserved sites. It has been suggested that STAT site clustering helps forming tetramers that co-operatively increase STAT transcriptional output (Bergad et al., 1995; Ota et al., 2004; Xu et al., 1996). Although STAT92E may form similar tetrameres in *Drosophila*, the distance of the clustered sites we observe here is probably too large to allow tetramer formation and their existence must serve another purpose. When we subdivide the *vvl1+2* enhancer we observe that the distal non-conserved STAT92E site is dispensable for the enhancer function (compare Fig. 5H and I). However mutagenesis of the two conserved sites in *vvl1+2* without mutating the distal non-conserved site has a mild decrease in the enhancer expression (Fig. 5C). Only when we mutate all three sites, including the non-conserved site, there is a strong effect on *vvl1+2* expression (Fig. 5D). This shows that non-conserved sites may be functional *in vivo* even though they are not absolutely necessary. Our results indicate that sites appearing distal to a STAT92E regulated enhancer may substitute for the proximal conserved sites, suggesting a way in which novel functional sites could eventually substitute conserved sites during evolution.

The *crb* and *dome* genes, which have been shown *in vivo* to be STAT92E targets (Lovegrove et al., 2006; Rivas et al., 2008), also show an accumulation of STAT92E sites in the introns where the enhancers localize. In the case of *dome,* five STAT92E sites cluster in a 700 bp fragment. Although only two of the five sites are conserved, and dissection of the enhancer showed that the conserved sites are crucial for JAK/STAT regulation, the non-conserved sites were also required for full enhancer expression (Rivas et al., 2008). Therefore, clustered conserved and non-conserved STAT92E sites contribute to target gene regulation. It will be important to understand how STAT92E proteins binding to these distant sites interact with other transcriptional co-factors that presumably bind to the core enhancer.

### STAT92E and the evolution of arthropod respiratory system

The earliest genes activated specifically in the tracheal primordia are *trh* and *vvl*. Both genes have cross-regulatory interactions that help maintain each other's expression in the trachea, but their early activation is independent of each other. Here we have localized the early trachea enhancers in *trh* and *vvl* and have shown that their activation in both cases depends on the JAK/STAT pathway making STAT92E the most important trachea activator. St10 *upd* expression is consistent with a model where *trh* and *vvl* activation in the tracheae primordia is specified by a competitive interaction between the JAK/STAT and the WNT signaling pathways. STAT92E is probably acting with some other transcription factor(s) as inactivation of the JAK/STAT pathway does not result in a complete lack of expression of the enhancers (Figs. 2H–I and 6E-G). These other transcription factors would not necessarily have a restricted spatial expression as the precise positional activation of *vvl* could be provided by *upd* and *wg*. Although the early requirement of STAT92E for early tracheal specification precludes any studies at later stages, the maintained expression of *upd* in later tracheal development and the presence of STAT92E conserved sites associated to late tracheal enhancers suggest that the pathway is important for *vvl* and *trh* expression maintenance during tracheal development.

The observation that in crustaceans *trh* and *vvl* are co-expressed in the epipods that form the gills suggest that both genes where co-opted early in arthropod evolution to control the formation of the respiratory system (Franch-Marro et al., 2006). It would be interesting to find out if JAK/STAT is also required for *trh* and *vvl* expression in the crustaceans as that would suggest that the pathway had been adopted early in evolution fixing the regulation of both genes in the respiratory system.
