# Supplementary material for: An efficient approach to isolate STAT regulated enhancers uncovers STAT92E fundamental role in Drosophila tracheal development
Source: Dev Biol. 2010 Apr 15;340(2):571–82. doi: 10.1016/j.ydbio.2010.02.015 (PMC2877871; doi:10.1016/j.ydbio.2010.02.015)
Supplement: Supplementary file 1 [file mmc1.doc]

**Supplementary table 1. Primers used to amplify *vvl* genomic DNA**

| **Primer name** | **5’ to 3’ sequence** |
| --- | --- |
| vvl1+2 fwd | ggttaatgatggccacacag |
| vvl1+2 rev | ccataatccagtttaatttccg |
| vvl1+2 s1rev | tcacacctacacagtgaaatgg |
| vvl1+2 s2 fwd | gctgcaattttccctggaaaaattacgtccagcacg |
| vvl1+2 s2 rev | atgatcctgttggcgccacag |
| vvl1+2 s3 fwd | cataaaatatgtagttatggg |
| vvl 0.9 fwd | cggaaattaaactggattatgg |
| vvl 0.9 rev | ccgcatgtcaaaaatgtcgc |
| vvl 345 fwd | gccgtccgattgatttttgtgcg |
| vvl 345 rev | cgcagcgccacccatgaaaaac |
| vvl 1.6/5.3 fwd | cctggacgttccataatcg |
| vvl 1.6/5.3 rev | acatgcgatcgattggcctc |
| vvl 6 fwd | aagaggccaatcgatcgcatg |
| vvl 6 rev | cccatttcagccagtgcgtg |
| vvl 1.8 fwd | acttggcttggttccccttc |
| vvl 1.8 rev | gccttgccaacctgtctcac |
| vvl9 fwd | gtttattgctgggctgggag |
| vvl9 rev | gcagccatcatcatatagaccg |
| vvl ds3.0 fwd | gttttttatttggggtttcg |
| vvl ds3.0 rev | caatcttttacatgtgggc |
| vvl ds 1.7 fwd | ggccacgccacataaagggc |
| vvl ds 1.7 rev | actatcctgctcgttccctcg |
| vvl ds 1.0 fwd | gttgaattcgatctccgggtgc |
| vvl ds 1.0 rev | gacgatatatagttctgaaaccc |
| vvl ds 1.5 fwd | ggagctgttaacactccttcagttagccc |
| vvl ds 1.5 rev | cgacacacggatcttccacgggg |
| vvl ds 0.5 fwd | ccttcttatcagcgatgctggcc |
| vvl ds 0.5 rev | cgagaaaatcttgtacgtgaccaaccg |

**Supplementary table 2. Primers used to amplify *trh* genomic DNA**

| **Primer name** | **5’ to 3’ sequence** |
| --- | --- |
| trh 24 fwd | gccccagcgttaggagttttccc |
| trh 24 rev | gaaaggtgatatgctgtgctcaagc |
| trh 31 fwd | cctgccggaagatctgaggc |
| trh 31 rev | gagaggtggagagcgaggg |
| trh 45 fwd | aaagtgcgagtcacgaatttgcc |
| trh 45 rev | ctatctttaattcaagcatgc |
| trh 47 fwd | cgcgcagtgtaggatatagttcc |
| trh 47 rev | tctacacctccccgaatttcc |
| trh 66 fwd | gctacaatccttacagtaaccttaatcg |
| trh 66 rev | atctccaatctagtacagattacc |
| trh 67 fwd | ttgacatcgattccaagctggagc |
| trh 67 rev | ctcgatatctcagtgtaagaggg |
| trh 75 fwd | ggtctgacacataaactaattctgc |
| trh 75 rev | gcagacgatttcaccactcc |
| trh 79 fwd | gaatgaaactgaaaatgggggtgag |
| trh 79 rev | cgtttcctgttggcctcccaccc |

**Supplementary table 3. Oligos used to mutate putative STAT92E DNA-binding sites in the *vvl1+2* enhancer.**

| **Oligo name** | **5’ to 3’ sequence** |
| --- | --- |
| Cons 3n Site 1 vvlS1 fwd | cgatacgga**ttc**tgc**gTT**gtcaccgggc |
| Cons 3n Site 1 vvlS1 rev | tgcctttgcccggtgac**AAc**gca**gaa**tccg |
| Cons 4n Site 2 vvlS2 fwd | gctgcaatt**ttc**cctg**gTT**aaattacgtccagcacg |
| Cons 4n Site 2 vvlS2 rev | cgtgctggacgtaattt**AAc**cagg**gaa**aattgcagc |
| Not cons 3n Site 3 vvlS3 fwd | ccacattagtcat**ttc**aaa**gTT**attaaagaaaactaaagtagaag |
| Not cons 3n Site 3 vvlS3 rev | cttctactttagttttctttaat**AAc**ttt**gaa**atgactaatgtgg |

STAT92E site in bold. Mutated bases in capitals.

**Supplementary table 4. Summary of vvl constructs**

| **Construct**  **(number of inserts studied)** | **Start**  **End** | **Embryonic expression** | **Number of STAT sites** | **Sequence** | **Conservation** |
| --- | --- | --- | --- | --- | --- |
| vvl1+2 | 3L:6757814 | Tracheal pits | 3 | TTCTGCGAA | Yes |
| (4) | 3L:6758493 |  |  | TTCCCTGGAA | Yes |
|  |  |  |  | TTCAAAGAA | No |
| vvl0.9 | 3L:6758472 | Oenocytes | 0 |  |  |
| (9) | 3L:6759436 |  |  |  |  |
| vvl345 | 3L:6759397 | Tracheal pits | 4 | TTCGTCGAA | Yes |
| (5) | 3L:6761285 |  |  | TTCAACGGAA | Yes |
|  |  |  |  | TTCCTGGAA | No |
|  |  |  |  | TTCATAGAA | No |
| vvl1.6/5.3 | 3L:6764932 | No | 0 |  |  |
| (7) | 3L:6766573 |  |  |  |  |
| vvl6 | 3L:6766551 | Specific | 2 | TTCCAGTGAA | No |
| (8) | 3L:6767816 | trachea cells |  | TTCGCTGAA | No |
| vvl1.8 | 3L:6770915 | Epidermis | 2 | TTCAGTGAA | Yes |
| (6) | 3L:6772795 | Late trachea |  | TTCTTTGAA | No |
| vvl9 | 3L:6774427 | PNS? | 1 | TTCTCAGAA | No |
| (3) | 3L:6775584 |  |  |  |  |
| vvlds3.0 | 3L:6797402 | Epidermis | 4 | TTCCAAGAA | No |
| (3) | 3L:6800370 | Late trachea |  | TTCGGTGAA | No |
|  |  |  |  | TTCATTTGAA | No |
|  |  |  |  | TTCATTGAA | No |
| vvlds1.7 | 3L:6810547 | Spiracles | 5 | TTCGTGGAA | Yes |
| (8) | 3L:6812238 |  |  | TTCGGTGGAA | No |
|  |  |  |  | TTCGTCGGAA | No |
|  |  |  |  | TTCAAACGAA | Yes |
|  |  |  |  | TTCGAGGGAA | No |
| vvlds1.0 | 3L:6816974 | Dorsal patches | 1 | TTCGATCGAA | Yes |
| (7) | 3L:6818083 | Epidermis |  |  |  |
| vvlds1.5 | 3L:6822486 | hindgut | 5 | TTCCGTGAA | No |
| (10) | 3L:6824044 | st11-st16 |  | TTCCTCGAA | No |
|  |  |  |  | TTCCGAAGAA | Yes |
|  |  |  |  | TTCATCGAA | Yes |
|  |  |  |  | TTCATTGGAA | Yes |
| vvlds0.5 | 3L:6841996 | No | 2 | TTCGTAGAA | Yes |
| (4) | 3L:6842495 |  |  | TTCGCTGAA | No |

**Supplementary table 5. Summary of trh constructs**

| **Construct**  **(number of inserts studied)** | **Start**  **End** | **Embryonic expression** | **Number of STAT sites** | **Sequence** | **Conservation** |
| --- | --- | --- | --- | --- | --- |
| trh24 | 3L:374245 | spiracles | 1 | TTCGTCAGAA | Yes |
| (4) | 3L:374665 | pharynx |  |  |  |
| trh31 | 3L:381340 | No | 3 | TTCCGATGAA | Yes |
| (6) | 3L:382840 |  |  | TTCAAATGAA | No |
|  |  |  |  | TTCATTGAA | Yes |
| trh45 | 3L:394668 | Late trachea | 2 | TTCGGGCGAA | No |
| (7) | 3L:395466 |  |  | TTCGCTGAA | Yes |
| trh47 | 3L:396509 | Tracheal pits | 4 | TTCGGCTGAA | No |
| (5) | 3L:398186 |  |  | TTCTATGGAA | Yes |
|  |  |  |  | TTCCGATGAA | No |
|  |  |  |  | TTCAATGAA | No |
| trh66 | 3L:415257 | Tracheal pits | 3 | TTCAAAGGAA | No |
| (5) | 3L:416768 |  |  | TTCCAGGGAA | No |
|  |  |  |  | TTCCAGAGAA | Yes |
| trh67 | 3L:416918 | Tracheal pits | 5 | TTCCCATGAA | Yes |
| (7) | 3L:418668 |  |  | TTCTCTGAA | No |
|  |  |  |  | TTCTCTGAA | No |
|  |  |  |  | TTCCCCGAA | No |
|  |  |  |  | TTCCTCGAA | No |
| trh75 | 3L:425468 | No | 2 | TTCAATTGAA | Yes |
| (6) | 3L:426068 |  |  | TTCAATTGAA | No |
| trh79 | 3L:428468 | No | 1 | TTCTAATGAA | Yes |
| (1) | 3L:429968 |  |  |  |  |
